# Dr. Mills on the Morbid Appearances in Disorders of the Trachea, Lungs, and Heart, &c.

**Published:** 1829-07-01

**Authors:** 


### An Account of the Morbid Appearances exhibited on Dissection in Disorders of the Trachea, Lungs, and Heart, with Pathological Observations, to which a Comparison of the Symptoms with the Morbid Changes has given rise. By *Thomas Mills*, M.D. Honorary Fellow of the King and Queen's College of Physicians. 8vo, pp. 302. 1829.

The attention which we have paid to the writings of Dr. Mills, proves our estimation of their merits, but we cannot help thinking that, like most of the Continental pathologists, he over-rates the value of morbid anatomy, in the study of diseases.

"The further I advance in this investigation, the more fully I am convinced that *morbid anatomy is the principal source of useful practical knowledge*; this enables us to trace the connexion between the sign and the morbid change; between cause and effect:—the cultivation of this branch can alone exalt medicine to the dignity of science." vi.

We believe it is the "principal source" of practical knowledge—but we are by no means convinced that "the cultivation of this branch can *alone* exalt medicine to the dignity of science." The farther we advance, and the more we see of diseases, the less hope do we entertain that medicine can even be a *science* in the common acceptation of that word. Where the same causes produce opposite effects—where we find the same symptoms with, and without, any organic change, how can we ever expect the certainty of *science* in physic? If indeed morbid anatomy always enabled us to "trace the connexion between the sign and the morbid change," we would fall down and worship it as the god of our idolatry;—but, knowing as we do that, in nine tenths of the diseases to which we are called, it is impossible to trace this connexion between symptoms and structural changes,—

for the good reason that either no morbid change exists, or, if it exists, it is incognizable by the senses—knowing this, we say, we can only look upon morbid anatomy as one of the paths that lead to the knowledge of diseases, and that by far the easiest one to tread. That morbid anatomy is not a *sine qua non*, is proved by many examples of good practitioners who knew hardly any thing of this branch. We need only instance Sydenham, who never alludes to the subject in his writings. In making these observations, we do not mean to deny that morbid anatomy lets in a flood of light on the causes as well as the effects of diseases—for all changes of structure must be regarded as the effects of previous disease, though they, in their turn, become the causes of other and still more serious maladies. When we consider how little power we have over the organic changes, (with the exception of mere inflammation) and the functional disturbances consequent on these changes, we have great need to watch the causes of the original disorders—the disorders themselves—as well as their consequences, the morbid structures. On these last Dr. Mills and the continental physicians seem to keep their eyes somewhat too steadily fixed—and yet the very existence of these "morbid changes" is a melancholy satire on medicine—for it is the great object, the great duty of the physician to cure *disorder*, and thus prevent *disease*. But to our author.

Dr. Mills observes, that a review of the cases and dissections detailed in this volume, leads him to the conclusion that the diseases, whether of the trachea, lungs, or heart, were of an inflammatory nature—and hence the danger of treating them, or any of them, as nervous or spasmodic.

"But let me not be misunderstood;—my object is the discovery of truth, and, while from facts I am compelled to maintain the inflammatory character of these diseases, I do not say that every disorder of the trachea, lungs or heart is inflammatory, nor am I an advocate for indiscriminate blood-letting; on the contrary, I would here repeat what I have already stated with respect to fever; in some cases it is useless or injurious, while others only require the use of diluents, aperients, a low regimen and confinement. In fine, though these diseases are essentially inflammatory, and though a rational mode of treatment must therefore be based on this principle, yet, in order to give efficiency to our practice, it is necessary to *individualize*,—that is, to consider the particulars of every case, as the age, sex, and constitution; the habits and previous ailments of the individual affected; moreover, to take into account the nature of the season and climate; of the prevailing epidemic, if any exist, and of the various influences, whether moral or physical, by which the patient is surrounded. Thus, while it necessary to *generalize*, in order to practise medicine scientifically; to practise it successfully, it is necessary to *individualize*." vii.

The work is divided into three portions—the first and shortest embracing cynanche trachealis or croup. To this we shall now direct attention.

### 1. Cynanche Trachealis.

There is no disease that demands prompter treatment than this. Dangerous in its tendency, and rapid in its progress, it may prove fatal in a day, or even in a few hours. Dr. M. has repeatedly met with cases of cynanche trachealis unaccompanied by that croupy noise in respiration by which it is usually distinguished. The practitioner, therefore, who waits for this supposed diagnostic symptom, may commit a fatal error.

"As far as my experience goes there is no such disease as spasmodic croup, or, croup unattended by inflammation—the contrary opinion appears to me pregnant with danger, as it leads the physician to trust solely to the employment of camphor, opiates, assafœtida, castor and other medicines called antispasmodic." 2.

### Cases.

1. The first case related by Dr. Mills, was one of acute cyn. trach. complicated with cynanche tonsill. supervening on measles. The patient was a boy, 5 years of age, who, after four or five days of feverish symptoms shewed the morbillous eruption, attended with a husky cough, which became alarming by being sometimes accompanied by a sense of suffocation and a croupy noise. The fever ran high—he was bled to six ounces—and had a black draught. Second day. The bleeding gave some relief—but the suffocating cough frequently returned. Fifteen leeches were applied to the fauces (as there was some difficulty of deglutition)—the warm bath was employed—and an emetic was exhibited. Third day. The symptoms not materially relieved. A blister to the throat—calomel and James's powder every three hours. *Fourth day.* Countenance swollen and livid—eyes staring—delirium, stupor, jactitation, pulse 130, skin hot and dry, speech inarticulate, appearance of strangulation, abdomen tense. Venesecteo ad ℥viij.—emetico-cathartic mixture. The bleeding produced instantaneous relief, and from this time the patient went on to convalescence.

### Comment.

"We have here a case of Cynanche Trachealis or Croup, accompanied by cynanche tonsillaris, which occurred during the eruptive stage of measles—must not this be ascribed to the inflammatory action of the vessels of the cutis having been communicated to the fauces, and thence to the larynx and trachea? The eruption was copious and the fever high; suffocation was twice threatened, and as often relieved by blood-letting, cathartics and emetics, and by the application of leeches and blisters near to the seat of disease. Calomel and antimonial powder were prescribed, and fomentations were employed without any obvious benefit." 6.

2. The second case which we shall select (being the 3d in the work) affords a melancholy proof of the rapid march of this dangerous disease.

"Miss W———, æt. 6, a lively, healthy child, was this morning attacked with a troublesome croupy cough attended with pain in the region of the trachea, with difficulty of breathing, fever, loss of appetite, languor and oppression; as the day advanced the breathing became more laborious, and the cough more frequent—the expectoration was scanty and mucous, the lips became livid, the face was alternately darkly flushed or deadly pale, the eye lost its lustre—about six o'clock p. m. when I was called on, the fits of coughing had become more violent and the respiration more oppressed, at times the child cried out that she was suffocating, and with an anxious and wild expression of countenance suddenly applied the hand to the neck.

"Death took place at ten o'clock, p. m. about twelve hours from the commencement of the attack.

"Dissection by Mr. M'Namara, assisted by Mr. Auchinleck. *April*, 1, 1823. The inferior portion of the trachea was covered with a thin cream-coloured purulent matter, its superior portion was completely obstructed by coagulable lymph, part of which had begun to put on the appearance of an adventitious membrane, the entire mucous coat had a preternaturally vascular appearance.

"Bronchiæ, lungs and heart, sound." 10.

Dr. Mills thinks that, had an emeto-cathartic mixture been given, and blood-letting employed early, together with the other antiphlogistic measures, this child might have been saved. Nothing was administered but a little castor oil before Dr. M. was called in, and then deglutition was impracticable. The dissection is satisfactory as elucidating the nature and seat of the disease.

3. The third case was one of chronic croup cured by the antimonial ointment. The patient was eight years of age, who complained of a feeling of pins and needles sticking in his throat, with hoarseness, cough, difficulty of breathing, and palpitation. The pulse was frequent and irregular, the face bloated, the eyes prominent and suffused. These symptoms had continued, more or less, for some weeks, but the croupy paroxysms were becoming more severe. Emetics, cathartics, and the hot bath had been employed with some advantage. Dr. Mills, however, prescribed leeches to the throat, and two grains of calomel every four hours. Considerable relief followed; but on the third day afterwards, there was a tremendous paroxysm, requiring an emetic and the tepid bath. Notwithstanding the depletory system, together with mercury, was fully employed for several weeks, the boy was harrassed with dangerous paroxysms of croup. At length a crop of pustules was brought out on the throat by the antimonial ointment, and the discharge kept up, by repetitions of the same. After this the boy had but one slight paroxysm, and went on to convalescence. The complaint lasted, in all, several months.

"In the mode of treatment" says Dr. M. "different remedies were employed at different periods; the most effectual during the paroxysm were blood-letting, emeto-cathartics, and the hot bath—active aperients were often required, and they always proved serviceable; from a combination of calomel and opium, much benefit was also derived; it was, however, to the use of the tartar emetic ointment that the disease finally gave way; this was applied to the external fauces, and the pustular eruption thus produced was great, and the subsequent discharge, copious; so great indeed was the inflammation and ulceration, that it became necessary to poultice for several days the external fauces:—since the application of the ointment there has been no return of the complaint, whereas, previously it returned every fortnight or three weeks:—it would appear then, as far at least as regards the case before us, that the counter-stimulus, and the long continued discharge thus excited, made a permanent impression on the disease, and totally changed the morbid condition and mode of acting of the internal vessels, which consequently resumed their wonted healthy actions,—now, as in three other similar instances, the same favourable result followed the use of this unguent, it may deservedly be ranked among the most valuable remedies for the cure of chronic croup, at the same time I am ready to acknowledge from experience, that like all others, it must, for obvious reasons, sometimes fail to produce the desired effect." 18.

In the commentary on another case which we do not deem it necessary to detail, Dr. M. observes that a spasm of the larynx or trachea, or of both, accompanies most cases of croup, and, in many instances, the danger is in proportion to its duration, and the degree of its intensity. "The spasms are induced by inflammation of the lining membrane of the wind-pipe, and then mildness or violence commonly depends on its degree and extent."

"To this general rule, however, there are exceptions, for in one post mortem examination, at which I was present, the marks of inflammation were not unusually striking, yet the spasms were urgent, and apparently caused the death of the patient.

"In the case now before us the spasms were violent, and often threatened suffocation ;—that they were caused by inflammation may be inferred from the good effects of evacuants and counter irritants." 22.

This portion of the volume closes with a case of cynanche maligna, which gives Dr. Mills an opportunity of advocating strong and active depletion. The sufferer in this case was a young lad 14 years of age, who was attacked with superficial ulceration of the tonsil, after exposure to cold, together with low fever. A mild aperient was prescribed, and not much attention was paid to the complaint ; but the ulceration gradually spread throughout the fauces, and assumed a livid appearance, with irregular edges, accompanied by fever, loss of appetite, and difficulty of swallowing. This was about the sixth or seventh day of the attack. On the ninth, there was acute pain under the sternum, with cough and difficulty of breathing—no amendment having taken place in the character of the ulcer.

"Ten ounces of blood were abstracted from the arm, the bark and wine were omitted, and a saline purgative was exhibited ; on the day following, the tenth of his illness, the chest was considerably relieved, the febrile symptoms gradually subsided, and from this period the ulceration in the tonsils began to assume a more healthy aspect : rubefacients were applied to the external fauces, the borax gargle was continued, and the aperient was frequently administered : under this treatment and a low regimen the patient was able to leave his room on the 18th day of the attack." 32.

It is dangerous to draw inferences from insulated cases ; but as far as an individual instance can be depended on, we agree with Dr. Mills that the amelioration in the appearance of the ulcer in the above case, after bleeding, would lead to the inference "that, under certain circumstances and in peculiar constitutions, cynanche maligna may demand the employment of blood-letting and aperients." Dr. M. properly adds that, in old and debilitated constitutions, or at the close of tedious illnesses, an opposite treatment will generally be necessary, viz. bark, wine, cordials, and nutritious diet—always remembering to keep the bowels open—and to apply leeches and blisters to the throat.

### II. Diseases of the Lungs.

Notwithstanding all that has been written of late, on pulmonary diseases, there will be found many important observations and facts in this part of our author's work. Dr. Mills is a practical man—and all facts recorded by such men are valuable.

On reviewing the cases and dissections contained in this section of the work, our author finds that ossification of the cartilages of the ribs, and chronic inflammation of the heart and lining membrane of the bronchia, were frequently detected in the bodies of those who had laboured under asthma—and that a collection of serous fluid in the pericardium often accompanied an obstruction of the lungs, even where the pericardium or heart was not diseased.

"In more than two or three cases where the right lung was extensively diseased, it has been discovered that pulmonary symptoms were mistaken for hepatic ; and, in one instance a disorder of the colon was mistaken for a disease of the liver. The detection of these errors is highly important ; it will induce us to pause a little before we decide on the seat or nature of the complaint, or prescribe a medicine which may possibly prove injurious." 38.

It would appear, indeed, that Dr. Philip's observations on hepatic and dyspeptic phthisis have blinded a great portion of practitioners to the existence of true pulmonary disease. Scarcely a day passes without our seeing people labouring under organic disease of the lungs, and yet under treatment for hepatitis or dyspepsia. If there be pain in the right side of the chest, it matters not in what part of the side, no doubt is entertained respecting the liver being the seat of the disease, and mercury is resorted to at once. If there be cough—it is stomach cough. If there be purulent expectoration—it is a sympathetic affection of the mucous membrane of the lungs, which requires no treatment, as the original malady is seated at a distance and must there be attacked! Meanwhile the disease goes on to a fatal termination, and if dissection shews the lungs disorganized and the liver sound, it was still hepatic phthisis, because Dr. Philip has clearly shewn that the original disease disappears in the progress of the symptomatic one, and consequently is not to be seen post mortem. We shall now proceed to some cases.

CASE 1. This was a gentleman, aged 42, corpulent, intemperate, and with an habitual appetite that was almost insatiable. When Dr. M. saw him he was complaining of cough and dyspnœa, with pain and a sense of weight in the left side, stretching towards the back. Pulse 104—tongue furred, yellow—thirst, restlessness. He was bled, blistered, purged; but four days afterwards, we find him with the same pain in the side—with dyspnœa, palpitation, cough, and purulent expectoration. He was again bled, blistered, and purged, with some trifling relief, and then sent to the country, where he did not stay long. In a few days he returned, complaining of "spasms of the lungs and legs," with numbness and weakness of the lower extremities. In about a fortnight he died.

*Dissection.* The colon was five inches in diameter, and its coats thickened. The stomach was five times its natural size, decending below the umbilicus. The spleen was of pulpy softness. Coats of the bladder thickened. Lungs hepatised in some places—bronchia lined with a thickish yellow fluid—some calculous concretions in the right lung—a whitish tumour, larger than an orange, of a semi-cartilaginous texture, containing numerous cells and tubercles filled with purulent matter, in the left lung. Four ounces of fluid in the pericardium—and about eight ounces in each side of the chest.

CASE 2.—VIOLENT ASTHMA. "Mr. S——, æt. 54, complains of cough, dyspnœa, palpitation of the heart, pain in the sternum, restlessness and extreme languor; tongue foul and yellow, pulse 110, strong; skin hot and moist; expectoration copious and purulent; bowels open; urine turbid. This gentleman is of a delicate frame, is sallow and emaciated, and, during the last ten years has been subject to frequent and violent fits of asthma, which terminate by copious gross expectoration." 47.

This was on the 2d of December, and the patient was bled to eight ounces. Next day he was nearly in the same state, and was purged and blistered. We need not go into the diurnal details. The patient died on the 23d of the same month.

*Dissection.* "Between the tunica arachnoidea and pia mater, a serous effusion is observable over the entire surface of the brain.

"The substance of the brain when cut into exhibits numerous red points.

"Nearly two ounces of a watery fluid are found in the ventricles.

"A quantity of serous fluid is discovered at the base of the cranium.

"Thorax, cartilages of the ribs generally ossified.

"Lungs, on both sides, adherent to the walls of the chest and to the mediastinum; and, on the left side, to the pericardium.

"Three small portions of calculous matter are found in the middle lobe of the right lung.

"No abscess nor ulceration is discoverable in any part of either lung; but there is a quantity of frothy purulent matter in the cells of the bronchiæ; the lining membrane of these cells is highly vascular.

"Pericardium,—contains about three drams of serous fluid; the internal surface of this membrane is rough, from a number of minute sabulous whitish particles dispersed through its texture.

"Heart,—substance of, natural, but its lining membrane is opaque in different points.

"The opening of the coronary vein is larger than natural.

"Liver,—natural in size and texture, but paler than usual.

"Gall-bladder, contains a thin olive-coloured bile.

"COMMENT.

"In this instance such was the violence of the pulmonary symptoms, that hydrocephalus, the immediate cause of death, was overlooked by the physicians in consultation, and the fatal event was supposed, previously to the dissection, to have been produced by a disease of the heart and lungs; a repetition of such mistakes can only be prevented by an examination of the body after death; this laid open the nature of the complaint, and showed, that our attention should have been directed to symptoms which, though only supervening to the original disorder, were the precursors of the death of the patient.

"Slight head-ach, confusion of ideas, and a sense of fulness in the head were complained of throughout the whole of the attack: a few days before death, the expectoration diminished, this was followed by high delirium, stupor, and the frequent application of the hand to the head, symptoms indicative of cerebral derangement, and produced by the accumulation of blood and serum in this organ; and, was not this accumulation the effect of congestion in the lungs, which, by obstructing the circulation, impeded the free return of blood from the head?" 53.

It appears that this gentleman had frequently recovered from similar attacks of the chest before—hence our author concludes that recovery might probably have taken place in the present instance, had not hydrocephalus supervened.

CASE 3. We shall notice this case, as it illustrates some remarks which we made a page or two back. The patient was a young lady 10 years of age, who, in January 1817, first complained of fugitive pains in the left side of the thorax and corresponding arm, accompanied by palpitation and sense of stricture about the heart, slight cough, and fever. These symptoms yielded to depletory measures—recurred, and yielded—till, at length, the attacks were frequent.

"In January and February, 1818, the pain in the region of the heart was often acute, and always attended with a sense of suffocation, palpitation, and at times, with purulent expectoration and hemoptysis. During the last three months the dyspnœa was permanent, often orthopnœa, and a sense of suffocation, accompanied by pains in the arms and shoulders; there were anasarcous swellings of the upper and lower extremities, and of the face; the tongue was aphthous, pains were frequently felt in the bowels, attended by a mucous diarrhœa; delirium and convulsions closed the scene.

"May 30th, 1818. *Dissection by Mr. Barker.* There is an elevation of the central portion of four of the ribs on the left side of the thorax, and, on this side the pleura pulmonalis and costalis, are adherent.

"The left lung is filled with tubercles and ulcers of different sizes, colours, and figures. Some of the tubercles contain a substance of a cheesy nature, others of a curdy or purulent, the majority are enveloped in distinct cysts.

"Pericardium,—considerably thickened; there is a deposition of coagulable lymph upon its outer surface, which is firmly attached to the diaphragm, in its cavity are found about six drams of a serous fluid.

"The heart is enlarged, and its surface highly vascular.

"The pleura, the left lung, the pericardium and the heart form one crude diseased mass.

"The right lung is less diseased than the left, but it contains two vomicæ; on this side, the pleuras are closely adherent.

"Liver,—left lobe, enlarged and stretches into the left hypochondre; structure natural.

"Stomach,—two large red patches, irregular in their size are found upon its lining membrane; one of which is situated between the cardiac and pyloric orifices.

"Intestines,—several portions of the small intestines are thickened and highly vascular, and on their mucous surface is observed puriform matter mixed with blood.

"Mezenteric glands,—enlarged, and some contain a cheesy and some a fatty matter." 57.

We are informed by Dr. Mills that, at the commencement of this young lady's illness, and for some time afterwards, "her complaint was considered hepatic, and mercurials were repeatedly exhibited; the pulmonary affection was regarded as secondary, and would, it was supposed, yield to the action of the remedies administered for the removal of the primary malady." Dr. M. avers that chronic inflammation of the bowels was here mistaken for disorder of the liver, and that "this mistake led to the use of medicines calculated to develop tubercles in the lungs, or inflame those which might otherwise have lain dormant." 60.

It is a melancholy reflection, and one calculated to lower our pride, that medicine should sometimes be the cause of mortal maladies when injudiciously administered for diseases not in themselves *fatal.* Yet we fear this is too often the case. Such a consideration ought to teach us to view with more charity and patience the ridiculed practice of some of our continental brethren—the "medicine expectante." Cases like that detailed by Dr. Mills, afford our neighbours fair game for retaliation. It is not, however, so much by activity of remedies that mischief is produced, where symptoms are urgent, as by mistakes in diagnosis. The young lady's case would hardly have been made worse by leeches, farinaceous food, and such means as are calculated to reduce inflammation, either in the chest or abdomen. But a false diagnosis being formed, wrong remedies were administered—and, at all events, the thoracic disease was neglected when it ought to have been the "all absorbing question." The study of auscultation will tend to correct this fatal error of diagnosis in future.

Case 4. Passing over two or three cases of no interest, we come to another instance of phthisis, mistaken in its early stage for hepatic affection.

We shall glance rapidly over the particulars. Miss D. aged 21, had been ill fifteen months. Pain in the right hypochondrium had been (as was said) the first symptom, to which succeeded cough, palpitation, dyspnœa, and hectic fever. " Two country physicians considering this a case of diseased liver, have given calomel in large quantity, and recommended a low diet, and latterly digitalis to abate the hectic." When Dr. M. was called in, there were cough, dyspnœa, perspirations, pains in both hypochondria, frequent and feeble pulse, emaciation, purulent expectoration. We need not detail the subsequent symptoms or treatment. The patient died in a couple of months, when the left lung was found tuberculous and filled with purulent or cheesy matter—right lung studded with tubercles loaded with pus and cheesy matter. The pericardium contained six ounces of water. The great lobe of the liver slightly adhered posteriorly to the parietes of the abdomen, and descended rather lower than natural; but was not altered in color or consistence. There was no other morbid structure in the abdomen.

" The dissection was instructive, as it served to correct an erroneous idea entertained with regard to the seat and nature of the complaint which was considered by some eminent practitioners as hepatic, and mercury was repeatedly administered, a medicine highly injurious in scrofula of the lungs.

" The pain felt in the right hypochondre gave rise to the opinion that the liver was the organ principally engaged, and the slight adhesion discovered between its convex portion, and the parietes of the abdomen indicated a low degree of inflammation that had subsisted at an early period of the attack, but the substance of the liver was sound, and the pain in the right side continued for months, this therefore must have depended on the disordered condition of the right lung." 75.

Two or three other cases and dissections are given, all shewing the fatal mistakes of practitioners on this particular point; but we shall pass them over. Our author gives us a kind of rambling essay on " Lymphatic Phthisis"—which is no other than what we call scrofulous or tubercular phthisis—epithets which he thinks objectionable, " because associated with the idea of debility, or of some undefined acrimony of the fluids," and thus giving rise to a " practice wavering and injurious."

" Were I allowed to form an opinion on this subject, grounded on observation and experience, I would say that the scrofulous tubercles of the lungs are lymphatic vessels, or a congeries of lymphatic vessels called glands in a state of inflammation and suppuration, consequently that the epithet lymphatic would be more appropriate, as it at once expresses the seat and nature of the disorder, and directs the practitioner to a rational mode of treatment." 124.

We confess that we do not see so very clearly how a certainty of the fact, that " scrofulous tubercles of the lungs were lymphatic vessels in a state of inflammation and suppuration," would lead us to a rational, or, at all events, to a successful practice. The inflammatory nature of phthisis, in its early stage, has been long advocated—and doubtless is true—but what have we gained by this knowledge when tubercles have arrived at suppuration? Very little we fear! We shall, however, allow Dr. Mills to speak for himself.

" Obscure in its origin and slow in its progress, lymphatic phthisis, is a disease alike insidious and dangerous, and often proceeds to an alarming height without exciting any serious apprehension; in our inquiry, therefore, we should not only take into account the present state but the past history of the patient, such as the healthy or diseased condition of the glands of the neck, axillæ and mezentery, the effects of dentition, vaccination, hooping-cough, scarlatina, measles, &c. and never lose sight of the state of the digestive and respiratory organs, for it is clear that visceral obstructions, by giving rise to congestion, irritation and inflammation of the lymphatic system frequently cause lymphatic or tubercular phthisis.

"We daily witness swelling, hardness, pain and redness of the lymphatic glands of the groin, in consequence of the irritation induced by blennorrhagia; the same symptoms arise from the irritation of a chancre on the penis or scrotum, or from the absorption of the syphilitic virus. Inflammation of the lymphatic glands of the upper parts of the legs, thighs and arms, proceeds from injuries or diseases of the feet and hands; the cervical glands often swell from the application of blisters to the nucha or head, and the axillary, from disorders of the mammæ, &c.; these facts are worthy of consideration, inasmuch as they show the excitability of the lymphatics, and the necessity of keeping this system of vessels in a healthy condition, for it is well known that pulmonary consumption is often preceded by swellings and inflammation of the lymphatic vessels and glands of the neck, axilla or thorax; a principal object therefore in the treatment of patients of irritable habits, or of those born of consumptive parents, is to diminish or remove this irritability, by giving tone to the constitution and preserving in a healthy condition the different organs and functions of life. Among the various remedies employed for this purpose, I shall now only observe that a light cooling and moderately nutritious diet is preferable to a full diet of animal food, jellies, soups, &c.; that wine is, for the most part, unnecessary, and often injurious by its stimulating qualities, and that the best remedies are such as are calculated to promote a healthy state of the secretions, particularly of the abdominal viscera.

"I have stated that lymphatic phthisis is obscure in its origin, and of slow growth, there are symptoms, however, by which its approach may be detected, and, in some cases, in time sufficient to arrest its progress,—these are, an appearance of delicacy in the expression of countenance, and in the frame of body, a slight cough, or rather hem, generally unattended by expectoration, and seldom noticed by the patient or his friends, and if noticed by strangers not acknowledged; the pulse is above the natural standard, and easily excited, the respiration is hurried by slight causes, and there is frequent palpitation; a face commonly pale or sallow, is easily flushed, a sense of weight or fulness is frequently felt in the head, accompanied by vertigo or tinnitus aurium, the usual exercises are followed by lassitude and languor, and there is a slow but gradual emaciation.

"As the disease advances, slight fugitive pains and a sense of oppression and weight are occasionally complained of in the chest and aggravated by a deep inspiration, the digestive organs are disturbed and the excretions assume a preternatural appearance, a low irregular fever comes on, indicated by thirst, heat of skin, slight chills, frequency of pulse and whitish tongue; such is a general outline of the complaint in its first stage, when there is some hope of checking its progress:—in its second or confirmed stage, the symptoms are so prominent and so dangerous, they can neither be mistaken nor removed; it is worthy of remark, that the sputa in tubercular phthisis, are seldom tinged with blood; these tubercles are found in distinct cysts, and being the product of diseased lymphatic vessels and glands, we cannot expect them to contain blood, but we discover a cheesy or curdy matter varying in color and consistence, according to the intensity and duration of the disorder, resembling the curdy whitish and wheyey matter detected in the lacteal glands of the mezentery when inflamed and suppurated." 127.

We shall give one more extract respecting climatorial and medicinal treatment.

"Among the means of prevention, setons or issues established in different quarters of the thorax, often prove highly serviceable, and in detecting some of the more early signs of this disease as well as the portions of the lungs most affected, considerable aid may be derived from the skilful application of the stethoscope, invented by the late scientific and distinguished Monsieur Laennec.

"Clothing sufficiently warm to protect the body from the vicissitudes of the weather is, at all times, necessary. A pure dry air, a serene sky, and an equable climate are often recommended, and no doubt, are very desirable; but, where are they to be found?—Every country has its disadvantages; in one, sultry heats and chilling dews; in another, intense cold; in a third, sudden vicissitudes of atmosphere; in a fourth, fogs, clouds, torrents, storms, or earthquakes; or we find that hot suffocating wind the Sirocco, or the cold piercing Bise, or that foul destructive air, the Malaria, or those pestilential vapours that arise from stagnant marshes; in short, whithersoever we go, we find some drawback—a something still wanting; why not then, especially when foreign travel and a foreign residence are impracticable, look to our own resources?—in them we may discover what will compensate us for the absence of the brighter skies of more southern latitudes. When the lungs are ulcerated and hectic fever sets in, foreign travel is worse than useless, it is injurious, for it subjects the invalid to cold, wet and fatigue, to many inconveniences and bad accommodation; and when we reflect on the want of almost every comfort in Southern Europe, on the absence of friends and society, and on the certainty of possessing these advantages at home, I do not hesitate to say, that in a well-regulated temperature of a large commodious dwelling, situated in the country, and protected from the easterly and north-easterly blasts, better health, more happiness, and longer life will be enjoyed than in France, Italy, Spain, Madeira, or in the isles of the Mediterranean." 129.

### Recovery from Phthisis.

We have now to look to the brighter side of the picture—a side which is too generally regarded with scepticism, or rather downright incredulity. Nevertheless, the light of modern pathology has shewn us that tubercular consumption is occasionally cured—and that, perhaps, by the unassisted efforts of Nature. This is proved by dissection, and there are many cases where we have good reason to believe in a cure, without the proof afforded by anatomy. We shall proceed to a cursory examination of those adduced by Dr. Mills.

Case 1.—Mr. ——, aged 32, of a pale, sallow complexion and spare habit, had been losing flesh, strength and appetite for ten months prior to the date of report, (19th October) while a dry teasing cough, attended sometimes with gross, purulent, flaky expectoration, evinced an affection of the chest. Hectic fever was now present—pulse 124, weak and irregular—skin dry—tongue whitish and tender—thirst—progressive emaciation—*pain in the right iliac region—bowels constipated—sense of heat, tenderness and distention in both hypochondria.* A blister was applied to the iliac region, and aperient pills were prescribed. 21*st Oct.* The iliac pain relieved—the cough troublesome—the expectoration purulent—fæces dark—urine lateritious. To take animal jellies; pills of rhubarb and ex. col. comp.; mucilaginous emulsion, with tincture of opium.

"*Oct.* 26*th.* Cough abated, expectoration not so copious; two or three dejections daily; fæces, at one time, scyballous, at another, indicative of vitiated bile; hectic fever diminished, better appetite. Cont. Med.

"Takes chicken for dinner and two glasses of claret.

"*Nov.* 6*th*. Expectoration less purulent and not so dark-colored; fever abated; feels some return of strength and spirits; is uncomfortable in his feelings unless he has two or three full dejections in the twenty-four hours; is obliged to increase the dose of the opening pills; complains of acidity of the stomach; lives in apartments heated to about 56°.—Fahrenheit.

"Pil. Cath. omni nocte.
Haust. ex rheo et magnesia
Omni mane.
C. Mist. anodyn. pro tussi.

"*Nov.* 13*th*. Skin now moist and of a more natural appearance; fæces more consistent, finds sensible relief after every evacuation; pulse 98; tongue cleaner; expectoration less copious and purulent:—takes two glasses of claret, animal jelly and some white meat daily,—complains of flatulence.

"Infus. amar. cum

Spir. Ammon. Arom.

C. reliqua.

"*Nov.* 20*th*. Is now able to read and to walk about his room for an hour without fatigue,—pulse 88. Cont. omnia.

"*Nov.* 24*th*. Cough and expectoration abated; two or three consistent dejections daily; urine lateritious; sleep refreshing; appetite and strength improving. Cont. omnia." 133.

From this time, the quantity of meat and wine was increased and the expectoration became diminished. On the 14th December, however, we find that the aperient had lost its effect—and that "*the cough became troublesome, the expectoration gross and copious, the pulse frequent, accompanied by diminished appetite and disturbed rest.*" "The aperient was changed; and, *as soon as the bowels were fully opened, the symptoms became moderate, and there was a restoration of strength and spirits.*" From this time, the patient went on to convalescence.

We have given the above case more fully than may seem necessary; but we consider it as a very unequivocal specimen of pulmonary affection, connected with, if not mainly dependent on, the abdominal derangement. The passages which we have specifically quoted, and especially those which we have marked in Italics, will, we think, carry conviction to the minds of our readers on this point. And here auscultation would have contributed much to the diagnosis between real and symptomatic phthisis—but the stethoscope was not employed. It is true Dr. Mills avers that, during his attendance, "tubercles were, at different times thrown up;—the cavities made by the discharge of so much purulent matter must have subsequently healed." We beg leave to doubt these conclusions, in the absence of any auscultic proofs in the living body, or anatomical demonstrations in the dead. Dr. M. is at a great loss to account for the good effects of purgatives in this case: but the difficulties vanish, if we come to the conclusion that the main seat of the disease was below the diaphragm. Dr. M. found, also, that small quantities of claret and nutritious diet, while they improved the strength and spirits, rather diminished than increased the hectic." The following case is still more in point.

Case 2.—This is headed as follows:—"A Case of Recovery from Phthisis Pulmonalis, in consequence of a Change from a low to a generous Diet, and from sedative Medicines to Wine and Cordials." The title is somewhat ominous to a former article of Dr. M.'s creed, namely, that all the cases treated of in this volume were "of an inflammatory nature." But to the case before us.

Mrs. ———, aged 49, is (Feb. 20) pale, weak, emaciated—sight imperfect—speech inarticulate—pulse feeble, frequent, and irregular—skin cool and constricted—tongue white—breathing oppressed. This lady has been living on a low diet and on tincture of digitalis for the cure of pulmonary consumption, "under which she has been labouring for many months." The sedatives were discontinued and were replaced by spiced wine.

"*Feb. 21st.* Was revived by the wine; cough not quite so troublesome; expectoration of a greenish-yellow; breathing less oppressed; no pain in the side or chest; pulse 120; some rest; body constipated; complexion sallow." 130.

Blue pill and colocynth—an opiate emulsion for the cough. Chicken broth and mulled wine. On the 26th, we find her improving on this plan, and three or four glasses of claret were allowed, in addition to animal food. On this system she gradually recovered.

The great imperfection in this case is, the total want of proof that the patient laboured under phthisis pulmonalis. The expectoration, indeed, at one time, is represented as "cream-coloured and sinking in water:" but that is a very inadequate proof of pulmonary consumption. We can spare room but for one more case, and that is headed—

"Hepatic Phthisis.

"Mrs. B———, æt. 21, of a sallow complexion, subject to scrofulous swellings of the neck, and born of parents who have often laboured under diseases of the liver, for three years has been subject to a slight cough, dyspnœa and palpitation, preceded or accompanied by fugitive pains in both hypochondres, occasionally shooting to the shoulders; or by a sense of weight or fulness in the right side; these symptoms have been attended by a low irregular fever, by acidity or flatulence of the stomach, by constipation and a diminished appetite; about a year ago an eruption of the furfuraceous kind appeared on the face, and within the last four months the affection of the chest has become more violent.

"Blisters, aperients, mucilaginous mixtures and anodynes have been prescribed.

"During the last week has complained of pain in the loins and head, of vertigo, loss of appetite and strength;—tongue yellow, fæces blackish, urine lateritious, pain in the right hypochondre and shoulder augmented by pressure, fever, troublesome cough, slight hemoptysis." 144.

Leeches—pil. hyd.—saline medicines. The mercurial treatment, with occasional leechings and purgations were continued, and partial recovery took place. The following is the commentary of the author.

"This is a case of hepatic phthisis, a formidable disease which requires at the onset a judicious and decided line of treatment; the complaint of the liver was prior to that of the lungs, and being obscure, gave rise to an alarming train of symptoms before it was fully developed or suspected; hence the teazing cough, hemoptysis and hectic fever, hence, the full display of chronic inflammation and enlargement of the liver with all its attendant symptoms of indigestion, and hence I fear the development of tubercles in the lungs. For the present, the patient is considerably relieved, but phthisis pulmonalis is ultimately to be apprehended. The cough, dyspnœa and palpitation which appeared early in the history of the case served to mark the lurking and primary disease of the liver, and, unfortunately, improper or frivolous remedies were administered, whereas, had just views been taken of the complaint at its commencement, and efficient means employed to arrest the progress of low inflammation of the liver and correct its morbid actions, have we not reason to say that the progress of disorder in the lungs might have been prevented? And this appears to be the useful practical question to which the case gives rise.

"On inquiry, I find, that this lady died in the county of Armagh of pulmonary consumption, in the year 1820, that is four years after the above illness." 146.

This brings us to the conlusion of the first half of the volume, embracing diseases of the trachea and lungs. The other half embraces affections of the heart, and will require a separate article. We accord to Dr. Mills the meed of zeal and industry—and although we do not carry our confidence in pathology to the same extent as he does, yet we venture to say that we attach to its study all the importance which it deserves. The value of some of the cases in this volume is evidently much diminished by the inattention to physical signs, where the nature of the complaint was not revealed by dissection. Dr. Mills is surely not too old to add to the stock of knowledge which he already possesses—and which is large—by the cultivation of mediate or immediate auscultation.

